# *Mycobacterium tuberculosis*-Associated Necrotizing Pneumonia With Adjunctive Corticosteroid Therapy

**DOI:** 10.1155/2019/9068516

**Published:** 2019-07-01

**Authors:** Ho Lam Nguyen, Huyen Duong Thanh, Thuong Vu Le, Ngoc Tran Van

**Affiliations:** ^1^Department of Internal Medicine, University of Medicine and Pharmacy at Ho Chi Minh City, Ho Chi Minh City, Vietnam; ^2^Respiratory Department, Cho Ray Hospital, Ho Chi Minh City, Vietnam

## Abstract

Necrotizing pneumonia induced by *Mycobacterium tuberculosis* is a rare but severe condition. It is difficult to distinguish between *M. tuberculosis*-associated and bacterial necrotizing pneumonia. The optimal treatment for this condition is controversial. Here, we report a case of *M. tuberculosis*-associated necrotizing pneumonia treated with the adjunctive corticosteroid and the antituberculosis drugs.

## 1. Introduction

Necrotizing pneumonia is a rare but severe condition of lung parenchymal destruction commonly caused by bacterial pathogens. Necrotizing pneumonia induced by *M. tuberculosis* was reported in children recently [1], and several published cases with *M. tuberculosis* pulmonary gangrene had been reported in adults before [2]. However, optimal therapeutic strategy for this condition is still controversial [3]. Here, we report an interesting case with *M. tuberculosis*-associated necrotizing pneumonia treated successfully with initiation of corticosteroid followed by antituberculosis drugs.

## 2. Case Report

A 44-year-old male patient was hospitalized because of progressive dyspnea, cough, and fever during one month. His past medical history was unremarkable. He appeared initially with mild fever, dry cough, and night sweating but untreated. After two weeks, he felt shortness of breath and coughed up yellow sputum. He had been admitted in the general hospital where he had undertaken the first bronchoscopy which revealed the negative results of bronchoalveolar lavage (BAL) for both acid-fast bacillus (AFB) smear and *M. tuberculosis* polymerase chain reaction (PCR). He had been diagnosed with community-acquired pneumonia but not improvement after a 10-day course of antibiotic therapy and was transferred to our tertiary hospital.

**Figure 1:**
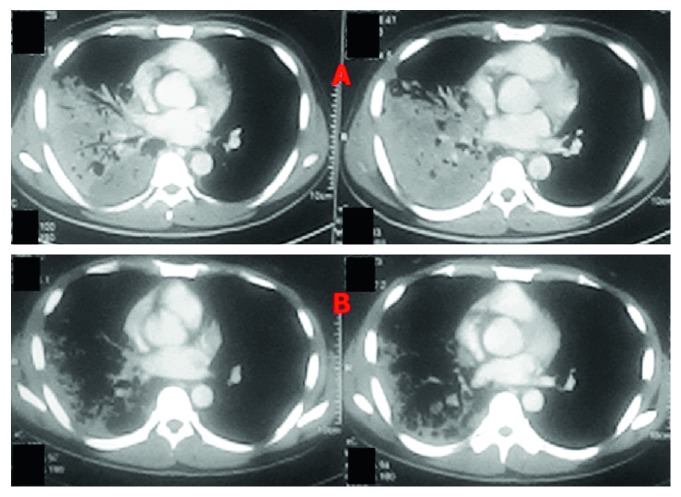
Two chest CT scans were undertaken 14 days apart. (a) The first chest CT showing consolidation at the right lower lobe with small cavities. (b) The second chest CT with significant amelioration of consolidation at the same lobe.

On admission, he was alert, with body temperature 37°C, pulse rate of 84 beats/min, respiratory rate of 28 breaths/min, and blood pressure of 120/80 mmHg. He was supplied with oxygen via bag-valve mask at 10 L/min, and the result of arterial blood gas showed pH 7.38, PaO_2_ 70 mmHg, PaCO_2_ 32.7 mmHg, and HCO_3_^−^ 19.4 mmol/L. Physical examination revealed dullness on percussion, fine crackles, and decreasing breath sound at the right lower lung field. Cell blood count with white blood cells 8.84 G/L (77.5% neutrophil and 13.3% lymphocyte) and hematocrit 38.2%, C-reactive protein level 78.4 mg/L, and mildly elevated liver transaminase level were recorded. The rapid testing for human immunodeficiency virus was negative. Chest X-ray showed consolidation in the right lower hemithorax, and the first contrast-enhanced chest computed tomography (CT) revealed small necrotizing cavities in this consolidation area (Figure 1(a)). With aforementioned information, we suspected differential diagnoses as follows: necrotizing pneumonia with particular pathogens (high-virulence bacteria, tuberculosis, or fungal infection) or noninfectious diseases (autoimmune disease, malignancy, or vascular disease). The antinuclear antibodies test was negative and the histopathological result of transthoracic lung biopsy showed an inflammatory process. He was treated with the combination of broad-spectrum antibiotics (meropenem, ciprofloxacin, and vancomycin) during four days but he still had fever. The adjunctive therapy with corticosteroid (40 mg methylprednisolone intravenous once daily) was commenced, and his condition improved spectacularly (defervescence, breath normally without oxygen) but the fever reoccurred on the fourteenth day after hospitalization. The second chest CT undertaken showed a significant regression of the consolidation (Figure 1(b)). The second bronchoscopy showed the positive BAL results for AFB smear and *M. tuberculosis* PCR. The treatment with a six-month antituberculosis regimen resolved his condition completely on follow-up.

## 3. Discussion

In our case, diagnosis of necrotizing pneumonia associated with *M. tuberculosis* was based on the consistent chest CT (lung parenchymal consolidation with lack of perfusion, microabscesses) and the positive BAL result for *M. tuberculosis*. The destruction of pulmonary parenchyma induced by *M. tuberculosis* usually develops from months to years in most cases. Nonetheless, there are a few cases (necrotizing pneumonia and pulmonary gangrene) in which this destruction may progress rapidly causing severe respiratory failure. The pathogenic mechanism can be explained by the intensive tuberculous inflammation causing the widespread vascular thrombosis and arteritis [2]. With this atypical manifestation of pulmonary tuberculosis, it is difficult to discriminate between *M. tuberculosis*-associated and bacterial necrotizing pneumonia [1]. According to the best of our knowledge, this is the first reported case of *M. tuberculosis*-associated necrotizing pneumonia in adults.

The adjunctive therapy of corticosteroid showed significantly improving outcomes of patients with not only severe community-acquired pneumonia [4] but also advanced pulmonary tuberculosis [5, 6]. Therefore, the corticosteroid treatment could be useful in management of *M. tuberculosis*-associated necrotizing pneumonia. Although the corticosteroid was initiated without antituberculosis drugs, the clinical and radiological tests showed significant improvement in our case. This can be through the beneficial effects of corticosteroid in reducing the intensive tuberculous inflammation. Moreover, the adjunctive therapy with corticosteroid should be considered in managing bacterial necrotizing pneumonia. However, using corticosteroids in these cases should be researched more before arriving at a definite conclusion.
